# Psilocybin therapy for treatment resistant depression: prediction of clinical outcome by natural language processing

**DOI:** 10.1007/s00213-023-06432-5

**Published:** 2023-08-22

**Authors:** Robert F. Dougherty, Patrick Clarke, Merve Atli, Joanna Kuc, Danielle Schlosser, Boadie W. Dunlop, David J. Hellerstein, Scott T. Aaronson, Sidney Zisook, Allan H. Young, Robin Carhart-Harris, Guy M. Goodwin, Gregory A. Ryslik

**Affiliations:** 1 COMPASS Pathways, London, UK; 2 Emory University, Atlanta, GA USA; 3 Columbia University, New York, NY USA; 4 Sheppard Pratt, Baltimore, MD USA; 5 University of California, San Diego, CA USA; 6 King’s College, London, UK; 7 University of California, San Francisco, CA USA

**Keywords:** Psilocybin, Depression, Natural language processing, Emotional breakthrough index, Machine learning, AI

## Abstract

**Rationale:**

Therapeutic administration of psychedelics has shown significant potential in historical accounts and recent clinical trials in the treatment of depression and other mood disorders. A recent randomized double-blind phase-IIb study demonstrated the safety and efficacy of COMP360, COMPASS Pathways’ proprietary synthetic formulation of psilocybin, in participants with treatment-resistant depression.

**Objective:**

While the phase-IIb results are promising, the treatment works for a portion of the population and early prediction of outcome is a key objective as it would allow early identification of those likely to require alternative treatment.

**Methods:**

Transcripts were made from audio recordings of the psychological support session between participant and therapist 1 day post COMP360 administration. A zero-shot machine learning classifier based on the BART large language model was used to compute two-dimensional sentiment (valence and arousal) for the participant and therapist from the transcript. These scores, combined with the Emotional Breakthrough Index (EBI) and treatment arm were used to predict treatment outcome as measured by MADRS scores. (Code and data are available at https://github.com/compasspathways/Sentiment2D.)

**Results:**

Two multinomial logistic regression models were fit to predict responder status at week 3 and through week 12. Cross-validation of these models resulted in 85% and 88% accuracy and AUC values of 88% and 85%.

**Conclusions:**

A machine learning algorithm using NLP and EBI accurately predicts long-term patient response, allowing rapid prognostication of personalized response to psilocybin treatment and insight into therapeutic model optimization. Further research is required to understand if language data from earlier stages in the therapeutic process hold similar predictive power.

**Supplementary Information:**

The online version contains supplementary material available at 10.1007/s00213-023-06432-5.

## Introduction

### Psilocybin and depression

Major depressive disorder (MDD) affects one in six adults in their lifetime, is characterized by at least one depressive episode with a duration of 2 weeks or more, and involves clear changes in mood, cognition, and the ability to experience pleasure (Otte et al. 2016). While MDD can often be effectively managed using psychotherapy and/or pharmacological treatments, a significant number of MDD patients do not respond to multiple treatment attempts. These patients form a distinct subgroup referred to as treatment-resistant depression (TRD) (Rush et al. 2006b; Otte et al. 2016).

Patients with TRD suffer severe hardship with an increased risk of impaired cognitive (Vancappel et al. 2021; Gregory et al. 2020) and social functioning (Judd et al. 2000; Hirschfeld et al. 2000; Thase and Howland 1994), reduced quality of life (Rathod et al. 2022; Lex et al. 2019), an increased risk of somatic morbidity (Lawrence et al. 2013), especially cardiovascular diseases (van Melle et al. 2004), and suicidality (Souery et al. 2007). In addition, the burden to caregivers, which is often overlooked, includes financial, emotional, physical, and social difficulties related to the care for the depressed person (Tabeleão et al. 2018; van Wijngaarden et al. 2004). Financially, the national cost of TRD in the USA has been estimated to be between $29 and $48 billion annually, assuming that 12–20% of all patients with depression have TRD (Mrazek et al. 2014). However, estimates of the prevalence of TRD vary widely in the literature (12–55%) (Wiles et al. 2014; Nemeroff 2007; Rush et al. 2006a; Corey-Lisle et al. 2002) and the actual costs might be higher. Existing options for next step treatment are various but often unsatisfactory leading to a high rate of relapse (Jain et al. 2022; Culpepper et al. 2015; Judd et al. 1998) or serious side effects such as sexual dysfunction, weight gain, and sleep disturbance (Ferguson 2001). The development of alternative therapeutic options for TRD patients with improved efficacy and acceptability remains an important and open challenge within healthcare.

Recently, therapies involving psychedelic treatment have shown positive results in addressing this challenge. Specifically, psilocybin, a tryptamine alkaloid (Passie et al. 2002), has shown potential as an antidepressant in preliminary studies involving patients with MDD (Davis et al. 2021; Carhart-Harris et al. 2021), TRD (Carhart-Harris et al. 2016), and anxiety in life-threatening cancer (Grob et al. 2011; Ross et al. 2016; Griffiths et al. 2016). More recently, the efficacy of COMP360 psilocybin therapy for TRD patients (at a 25 mg dose), was demonstrated in the first adequately powered multi-site, randomized, double-blind phase IIb study (Goodwin et al. 2022). MADRS score change at week 3 was the primary outcome of the study though a large reduction in depressive symptoms was evident on the day following psilocybin dosing and a subsequent durable response was observed in 20% of the 25 mg group at week 12.

As part of the COMP360 treatment, psychological support was provided to ensure the participants’ physical and psychological safety and consisted of three phases: preparation, administration, and integration. In the preparation phase, the participant met with a qualified therapist at least three times in order to receive psychoeducational material, prepare for the psychedelic experience, and build trust. During the psilocybin administration session, typically lasting 6–8 h, the same therapist was present and participants were encouraged to attend to their full range of internal experiences. Subsequently, during the integration sessions, the participants were encouraged to reflect on their experiences and any emerging insights (Tai et al. 2021). These integration sessions were lead by the same therapist who guided the participant through preparation and the psilocybin administration session.

Here we employ a novel measure of the average linguistic sentiment scores of the participant and therapist computed from transcripts of the integration session 1 day after psilocybin administration, together with items from the participant’s self-reported Emotional Breakthrough Inventory (EBI) and the participant’s psilocybin dose, to predict enduring responses at 3 and 12 weeks post psilocybin administration. Sentiment, specifically valence and arousal in speech, reflects mood states and has been implicated as a classifier of depression (Stasak et al. 2016). Similarly, EBI scores, which are believed to index experiences of emotional release or catharsis, have been shown to predict outcome in a recent psilocybin trial as well (Roseman et al. 2019). Our hypothesis was that the NLP and EBI information collected during the integration session would predict the primary outcome (MADRS scores) measured at 3, 6, 9, and 12 weeks after the psilocybin administration session.

## Methods and materials

### Study overview

COMP001 was a phase IIb, international multicenter randomized, double-blind study of COMP360, which randomized 233 adults with TRD in 1:1:1 ratio to a single fixed-dose (25 mg, 10 mg, or 1 mg) of COMP360 that was administered with a well-defined psychological support model that emphasizes safety and self-directed enquiry (Tai et al. 2021). The primary efficacy endpoint was change from baseline at week 3 in the Montgomery-Åsberg Depression Rating Scale (MADRS) total score. The primary study results and the details of the protocol have been previously published (Goodwin et al. 2022).

#### Study participants

The analysis was restricted to the clinical trial participants whose sessions were conducted in English and the therapists who provided psychological support to those participants. We note that all therapists were licensed mental healthcare professionals specially trained in the COMPASS psychological support model (Tai et al. 2021), but were not necessarily certified in clinical psychology. As per the study protocol, participants had the option to opt-out of recording their psychological support sessions. From the 233 participants enrolled in the main study, 185 consented to having their psychological support sessions recorded, 107 of these integration sessions were conducted in English. Five of these participants were excluded from the present analysis due to not having a MADRS score recorded at week 3, and one was excluded due to having a baseline MADRS score that was in the normal range, resulting in 101 participants for the week 3 target outcome prediction. Of these, 11 were missing MADRS scores at 6, 9, and/or 12 weeks, leaving 90 participants for that analysis. More information about both clinical trial participants and the therapists providing the psychological support can be found in Goodwin et al. (2022).

#### MADRS outcome measure

For each trial participant, seven MADRS scores were collected by a remote blinded rater: at baseline, the day after administration, and then at 1, 3, 6, 9, and 12 weeks post-administration. Here we focus on the change from baseline at week 3, and sustained change from baseline through week 12.

A *responder* was defined as a participant whose MADRS score at week 3 post treatment was reduced by at least 50% relative to that participant’s baseline MADRS score and a *sustained responder* was defined as a participant with at least a 50% reduction in MADRS score at each of weeks 3, 6, 9, and 12 after treatment.

### Predictive model

Our goal was to predict, with a logistic regression model (see Supplementary Material §2 for details on the model fitting), which participants would be week 3 responders and sustained responders through week 12 using information available immediately after treatment. This included NLP metrics from the transcript of the first integration session post psilocybin administration (i.e., 1 day after the administration session), items of the EBI that were measured at the same integration session, and the treatment dose.

The NLP pipeline begins with an audio recording of the integration session following psilocybin administration and ends with four numbers: two to summarize the average sentiment (valence and arousal) of the utterances spoken by participant, and two corresponding numbers for the therapist. Key steps in this process include:

- The creation of the audio recording,
- The transcription of recordings into text,
- Parsing the text into utterances,
- Computing sentiment scores for each utterance,
- And computing session averages of sentiment values.

We describe these steps in detail in the Supplementary Material, see §1.

#### Integration session sentiment

All psychological support sessions were audio recorded with an Apple iPhone (see Supplementary Material §1.1 for full details on audio recording). The audio recordings of the integration sessions were manually transcribed into dialog text, and each of these transcripts was then parsed into individual “utterances” (see Supplementary Material §1.2 and §1.3 for transcription and utterance parsing details). These utterances were used to estimate session sentiment for the therapist and participant using a novel sentiment model based on a large-language model that was inspired by the two-dimensional scale of emotion described in Russell (1980). Specifically, this model produces valence and arousal scores for each utterance. The valence score corresponds to the positive (pleasantness) or negative (unpleasantness) score typically used in sentiment analysis. The arousal score characterizes the physiological arousal expressed in the utterance and indicates where it lies on the spectrum from bored/calm to tense/alert/excited (see Supplementary Material §1.4 for utterance sentiment, §1.4.1 for the sentiment model and §1.4.2 for the session sentiment score).

#### Large language models

To compute our sentiment valence and arousal scores, we used a zero-shot classifier built on the BART autoencoder and the MNLI language inference dataset (Lewis et al. 2020; Yin et al. 2019; Williams et al. 2018). The classifier is available on the Hugging Face website https://huggingface.co/facebook/bart-large-mnli/tree/main.

#### Emotional breakthrough inventory

The EBI is an eight-item questionnaire introduced by Roseman et al. (2019) which can predict change in wellbeing following psychedelic therapy (Roseman et al. 2019). The EBI summary score is typically computed by taking the average of the eight items. However, we found that summarizing the EBI with a weighted average of the eight items based on the first component of a principal component analysis (PCA) performed slightly better than the simple average (see Supplementary Material §2.1 for details), and thus used the weighted average for our analysis.

## Results

### Logistic regression model fits

Before exploring cross-validated predictions for the two outcome measures, the logistic regression models created (see Supplementary Material §2.2) were fit to all the data to confirm that the models resulted in an adequate fit. The pseudo *R*-square values and chi-square significance test for each of the models are shown in Table 1. These results suggest that the models are well-fit to the data and worthy of further evaluation.

**Table 1:** Logistic regression model fit summaries

Model	Pseudo *R*-squared	Chi-square	*p*-value	df	*N*
Week 3	0.514	64.78	1.66e$-$11	7	101
Sustained	0.435	39.18	1.8e$-$06	7	90

**Fig. 1:**
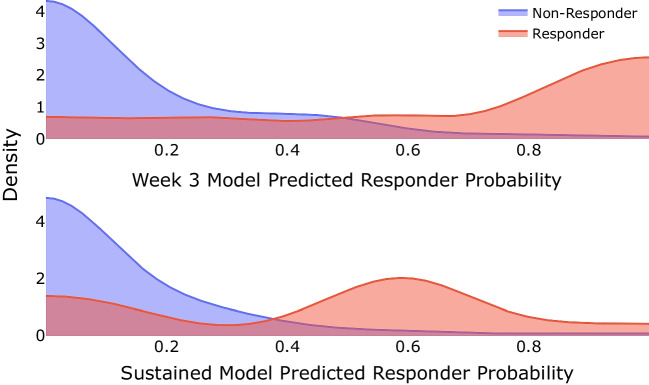
Model fit probability distributions estimated using Gaussian kernel density estimation for the week 3 model (top) and the sustained responder model (bottom). We note that the distributions are quite different between the non-responder and responder participants. This difference is used to successfully classify patient response status as shown in §3.2

Logistic regression models predict class membership (responder vs. non-responder) probability for each subject. It is useful to inspect the distributions of these class probability predictions to evaluate class separability; the distributions for the models are shown in Fig. 1 and suggest that the two classes should be quite separable.

### Cross-validated predictions

**Fig. 2:**
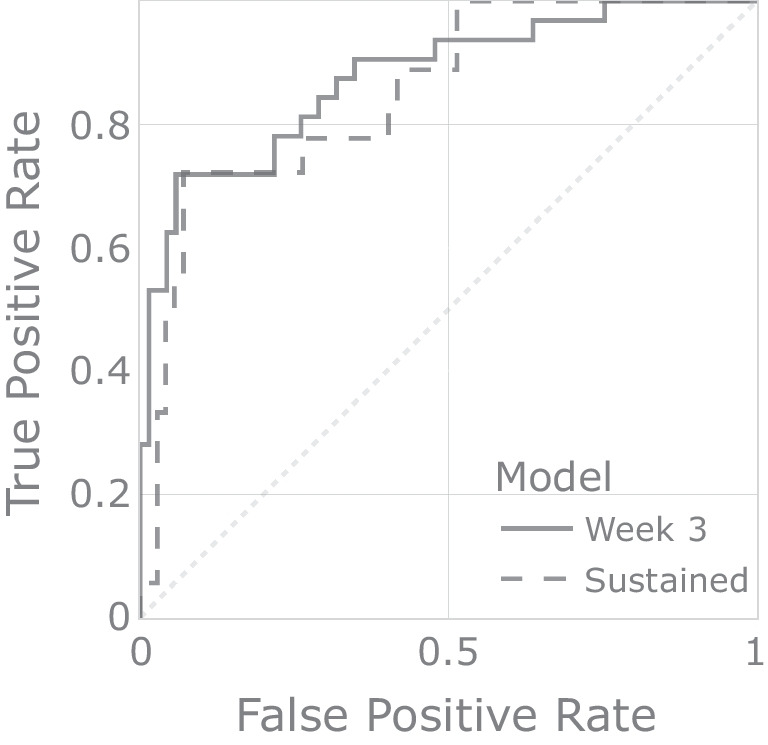
Receiver operating characteristic curves for the week 3 and sustained responder model predictions

The results from the leave-one-out cross-validation for the models described above are shown in Table 2. Leave-one-out cross-validation simulates the case where a model that was trained on existing data is used to predict the responder status of a new participant, and is thus ideal for estimating the real-world performance of a predictive model (see Supplementary Material §2 for details). For each model, results are shown for all participants together, as well as for each of the three treatment groups. A simple bootstrap was also used to compute 95% confidence intervals for the AUC values, which are shown in Table 3. The full receiver operating characteristic plots for these predictions are shown in Fig. 2.

**Table 2:** Cross-validated model prediction results, where MCC is the Matthews correlation coefficient and f1 is the harmonic mean of precision and recall

Model	Group	Accuracy	MCC	f1	AUC	TN	FP	FN	TP	*N*
Week 3	ALL	0.851	0.650	0.754	0.877	63	6	9	23	101
Sustained	ALL	0.878	0.610	0.686	0.853	67	5	6	12	90
Week 3	25 mg	0.821	0.641	0.829	0.886	15	3	4	17	39
Sustained	25 mg	0.833	0.639	0.769	0.880	20	3	3	10	36
Week 3	10 mg	0.935	0.746	0.750	0.862	26	0	2	3	31
Sustained	10 mg	0.963	0.693	0.667	0.760	25	0	1	1	27
Week 3	1 mg	0.806	0.380	0.500	0.820	22	3	3	3	31
Sustained	1 mg	0.852	0.250	0.333	0.722	22	2	2	1	27

**Table 3:** AUC values with bootstrapped lower and upper 95% confidence intervals

Model	AUC	Lower	Upper
Week 3	0.877	0.794	0.946
Sustained	0.853	0.747	0.943

### Univariate scatterplots

**Fig. 3:**
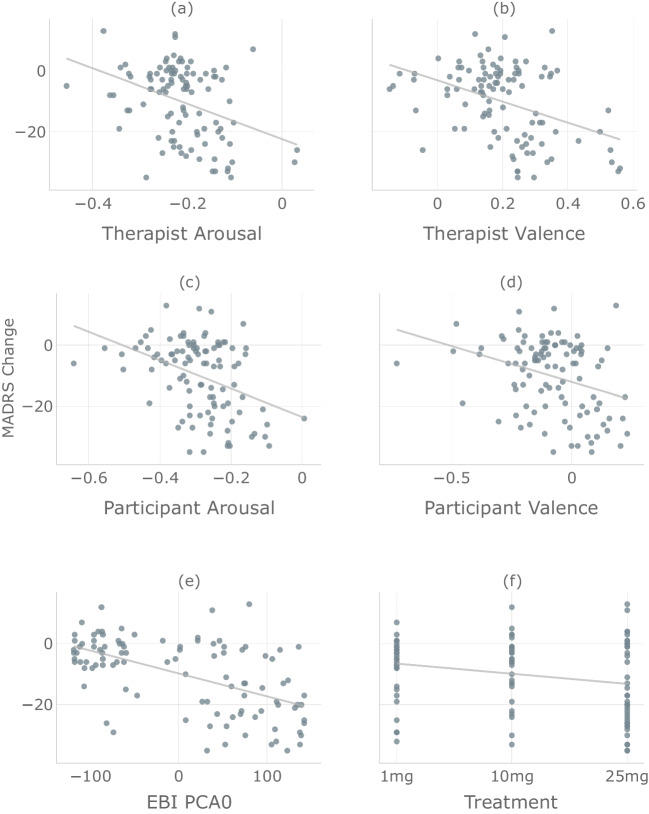
The relationship between the six predictors and week 3 MADRS change ($n=101$) is shown for therapist arousal (**a**, $r=-0.370$, $p=0.0001$), therapist valence (**b**, $r=-0.407$, $p=2e-05$), participant arousal (**c**, $r=-0.397$, $p=4e-05$), participant valence (**d**, $r=-0.322$, $p=0.001$), EBI summary score (**e**, $r=-0.556$, *p *= 2e-09) and treatment group (**f**, $r=-0.230$, $p=0.02$)

To further understand the relationship between each of the exogenous variables in the predictive models described above and the MADRS scores, we plot each of these variables against the MADRS week 3 change. These scatter plots are shown in Fig. 3, along with a regression line. All six variables are negatively correlated with the week 3 MADRS change, meaning that a higher value on the exogenous variable is associated with a greater MADRS reduction at week 3. This pattern is consistent with the regression model coefficients being generally positive (i.e., higher scores are associated with a higher probability of being a responder). The results are also consistent for the exogenous variables with agreed upon interpretations. For example, as the EBI PCA summary score increases, the MADRS reduction, as expected, increases as well.

## Discussion

For individuals undergoing psilocybin therapy for TRD, response outcomes at 3 and 12 weeks post-dosing could be predicted using the EBI measure or with NLP methods, achieving high accuracy with AUCs ranging from 85 to 88%.

The quality of the interaction between participants and therapists has been shown to be a mediating factor in overall outcome of psilocybin therapy for depression by facilitating a stronger emotional response (Murphy et al. 2022), and the NLP metrics described here capture key aspects of this interaction as it relates to the emotional response. Therapists trained in the COMPASS psychological support model encourage participants to engage with their emotional experiences in the present moment. Engagement with these experiences (without attempting to avoid or control them) is thought to decrease anxiety and enable the participant to have a more therapeutic COMP360 experience. As such it is believed that a stronger emotional experience, as measured by EBI and the NLP metrics, may underlie the basis for sustained response to COMP360 treatment. However, we note that while therapists were trained and evaluated in this psychological support model, the lack of fidelity metrics precludes us from evaluating the impact of adherence on outcome and suggests this is a ripe topic for future work.

Moreover, the four NLP metrics and EBI perform about equally well when each is used alone in a partial model (see Supplementary Material §2.3) and statistical performance is improved only moderately when EBI and NLP features are combined into the same model. This suggests that the therapeutic effects may be driven by the same latent variable. Our interpretation is that the fundamental mechanism of COMP360 treatment is to drive emotional breakthrough and a more positive integration session sentiment, which produces a more productive, psychologically insightful session (Peill et al. 2022), and thus results in a positive therapeutic outcome.

We note that the algorithm described in this paper is very fast to train and execute because it relies only upon logistic regression and PCA. As such, a new model may be fit on a standard consumer laptop within seconds of new data becoming available. Moreover, with modern cloud architecture, the transcription and sentiment analysis that forms part of the input to this ML model can be computed in real time. Taken together, this allows model updates to occur at whatever cadence is deemed desirable and allows physician insight into the participant’s ultimate response to the treatment immediately after the participant’s integration session instead of weeks to months later.

Finally, it is striking that NLP alone gives such a good measure of immediate and early drug effect without recourse to subjective rating scales or even the EBI. It suggests key elements of the participant’s mental state can be captured by a passively measured behavioral measure in real time. By utilizing similar information from the preparation and drug administration sessions, we may be able to identify additional signals that improve the accuracy of our predictive model. Furthermore, by using NLP signals from preparation sessions alone, it may be possible to identify which participants are likely to be more responsive to COMP360 therapy prior to the drug administration session. Preparedness may be an important variable for selecting patients for COMP360 therapy, and possibly other psychedelic treatments (Haijen et al. 2018). Additionally, we see future potential for this approach to offer insight into the quality of the psychological support that we assume to be essential for the safe delivery of psychedelic treatments. NLP analysis of the preparation and integration session dialog may also shed light on how treatments may need to be adapted or improved for those who do not respond to an initial treatment.

Limitations of this study include that we were only able to analyze a cohort of 101 participants at 3 weeks and 90 individuals at 12 weeks. While this sample size is more than sufficient for the logistic regression models used in this study, additional subjects from future studies will allow us to further validate the current findings and utilize more advanced machine learning models for prediction such as random forests (Breiman 2001) and neural nets (Mikolov et al. 2013; Vaswani et al. 2017; Devlin et al. 2018; Brown et al. 2020) while reducing the risk of overfitting. Also, because all therapists were trained to follow a specific, well-defined, and non-directive psychological support model (Tai et al. 2021) and not all of the therapists were clinical psychologist, the results may not generalize to other psychedelic therapy contexts. Finally, the burgeoning field of digital biomarkers (Coravos et al. 2019; Jacobson et al. 2019; Cavedoni et al. 2020) may also be integrated to provide signals that correlate with participant response before, during, and after treatment.

In summary, recent advances in utilizing psychedelic treatment for participants with depression show promising results (Goodwin et al. 2022; Davis et al. 2021; Muttoni et al. 2019). In this paper, we demonstrate that we are able to accurately predict treatment response to COMP360 in combination with the associated psychological support model (Goodwin et al. 2022; Tai et al. 2021) using a logistic regression machine learning model that includes NLP metrics from the first integration session, participant’s self-response on the EBI scale, and treatment arm. In turn, this ability to predict outcomes has substantial beneficial implications to providers, payers, and participants with respect to future additional treatment or interventions. As additional language and EBI data are collected and combined with digital biomarkers from tools such as smart devices, we expect that this methodology, along with more powerful machine learning models, will further increase our ability to predict participant response to psychedelic treatment. Ultimately, this will allow for more objective, real-time, and personalized care for future patients.

## Supplementary Information

Below is the link to the electronic supplementary material.

Supplementary file 1 (PDF 430 KB)

## Data Availability

Data and code necessary for reproducing the figures here can be found at https://github.com/compasspathways/Sentiment2D.
