# Supplementary material for: Psilocybin therapy for treatment resistant depression: prediction of clinical outcome by natural language processing
Source: Psychopharmacology (Berl). 2023 Aug 22;242(7):1553–61. doi: 10.1007/s00213-023-06432-5 (PMC12226623; doi:10.1007/s00213-023-06432-5)
Supplement: Supplementary file 1 — (PDF 430 KB) [file 213_2023_6432_MOESM1_ESM.pdf]

Supplemental Materials for "Psilocybin Therapy for Treatment Resistant Depression: Prediction of Clinical Outcome by Natural Language Processing"

1 NLP Pipeline

The natural language processing pipeline begins with an audio recording of the integration session following psilocybin administration, and ends with four numbers which capture the average sentiment of the utterances spoken by participant and therapist during the session. The key steps in this process are described below.

1.1 Audio Recording

Prior to the psychological support sessions, the therapists obtained written consent from participants to record the sessions. All psychological support sessions were audio recorded with an Apple iPhone using the built-in microphone and Voice Memo application. Each device was pre-configured with a secure cloud storage account. Once the recordings were completed they were automatically uploaded to the configured storage account. After quality assurance checks to ensure the metadata for each recording was accurate, the recordings were made available for transcription.

1.2 Transcript Production

All audio recordings were manually transcribed by a clinical research services vendor (Illingworth Research Group). Identifiable information of both the participant and therapist were removed to anonymize the transcripts. A number of transcripts were randomly selected and double checked by independent transcribers to ensure transcription quality and anonymization. After these quality assurance steps, the transcripts were made available for analysis.

1.3 Utterance Parsing

After converting the audio recording into text, each transcript was parsed into individual “utterances” using the transcript punctuation as a guide. The primary reason for doing this was to leverage an existing pre-trained “large language model" for our

sentiment scoring (described below in §1.4.1). This model was designed to analyze individual sentences, and so breaking our transcripts into sentence-like pieces facilitated its use. Ultimately, the goal was to be faithful to the original spoken content, while producing text as familiar to the pre-trained model as possible.

Typically in NLP, an utterance is defined to be a spoken group of words that is preceded by and followed by a pause. In contrast, a sentence is a group of words that expresses a complete thought. Our "utterances", having first passed through the transcription process are an amalgamation of the two. For instance, if a sentence occurs over several utterances, we combine the pieces and form a single "utterance", or if an utterance contains several sentences, we extract each sentence and treat them as stand-alone "utterances".

Additional processing steps that were taken after transcription include: converting Unicode characters to ASCII characters, replacing redacted information such as names with a non-personally identifiable alternative (e.g. Jane/John Doe), and replacing transcriber comments with model-familiar text (e.g. a transcriber's note "[LAUGHING]" became "haha").

1.4 Utterance Sentiment

Sentiment analysis of a piece of text is the practice of scoring it as positive or negative. We took a nonstandard approach that scored text in two dimensions, "valence" (aka "positivity"), and "arousal" ("energy" or "activation"). This was inspired by work in psychology on two-dimensional affect, and was motivated by our desire to find a way to score sentiment that captured intensity, rather than just positivity or negativity.

The sentiment score of a piece of text is related to but different than trying to infer the emotional state of the speaker. For instance, "I love broccoli" would likely be scored by most sentiment models as positive, however if vocalized in a sarcastic way it would signal a negative attitude towards broccoli.

Our initial experiments with sentiment models were disappointing because we found that scores did not capture intensity. For example, "I love broccoli" (97% likely positive in one model) and "I like broccoli" (98% likely positive in the same model) would receive essentially the same positivity score, even though we felt "love" should carry more strength than "like". We suspect that this is because a sentiment problem is typically treated as a classification problem, and thus intensity is irrelevant—all that matters is getting the "positive" or "negative" label correct.

Our solution took inspiration from the literature on two-dimensional models of emotion that were introduced by Russell under the name "The Circumplex Model of Emotion" [1]. A two-dimensional model plots an emotion in the xy-plane where the x-value represents **valence** (i.e., "positivity") and the y-value represents **arousal** (i.e., "energy" or "activation"). Examples of high valence emotions are "giddy","happy", "content", and "serene". Low valence emotions include "fear", "nervousness", "sadness", and "boredom". High arousal emotions include "tense", "alarmed", "astonished", and "excited". Some low arousal emotions are "bored", "droopy", "tired", and"sleepy".

1.4.1 The Sentiment Model

To compute our sentiment valence and arousal scores, we used a zero-shot classifier [2] built on the BART autoencoder [3] and the Multi-Genre Natural Language Inference (MNLI) dataset [4]. The use of zero-shot models built on top of large language models is a powerful way to bring vast amounts of freely available data to bear on “small data" tasks.

BART is a deep learning model originally designed to reconstruct text that had been corrupted. It was trained on an enormous corpus of books and wikipedia content. The result of fine-tuning BART on the MLNI dataset is a “zero-shot" classifier for any given set of classes. This means, the user can specify a list of classes, and then submit a piece of text to be classified. For instance, “I love broccoli" could be submitted to be classified into the classes "food" and “politics". The model thinks there is a 99.8% chance this text is about food, but allows for a 0.2% possibility that it is about politics.

Due to the enormous amount of pretraining BART has before learning the classification task, the classifier has proven to be amazingly powerful `https://joeddav.github.io/blog/2020/05/29/ZSL.html`. The classifier is available on the Hugging Face website `https://huggingface.co/facebook/bart-large-mnli` [5].

In our application, for each utterance we used the model to score the likelihood that it should belong to one of the following four classes: "happy", "angry", "gloomy", and "calm". For an utterance u, the model provides the scores

$$\mathrm{P}_u(\text{“happy"}), \mathrm{P}_u(\text{“angry"}), \mathrm{P}_u(\text{“gloomy"}), \text{ and } \mathrm{P}_u(\text{“calm"}) \tag{1}$$

These are positive numbers which sum to 1.

In order to use the scores to plot a point in the two-dimensional emotional plane, we assigned valence-arousal pairs to the four classes

$$\begin{array}{lll} \bullet\ \text{“happy"} & = & (\ \ 1,\ \ 1) \\ \bullet\ \text{“angry"} & = & (-1,\ \ 1) \\ \bullet\ \text{“gloomy"} & = & (-1,-1) \\ \bullet\ \text{and “calm"} & = & (\ \ 1,-1) \end{array} \tag{2}$$

and then defined the point $(\text{valence}(u), \text{arousal}(u)) =$

$$\begin{aligned} &\mathrm{P}_u(\text{“happy"})(\ \ 1,\ \ 1) + \mathrm{P}_u(\text{“angry"})(-1,\ \ 1) \\ &\qquad\qquad + \mathrm{P}_u(\text{“gloomy"})(-1,-1) + \mathrm{P}_u(\text{“calm"})(\ \ 1,-1). \end{aligned} \tag{3}$$

Equivalently,

$$\text{valence}(u) = \mathrm{P}_u(\text{“happy"}) - \mathrm{P}_u(\text{“angry"}) - \mathrm{P}_u(\text{“gloomy"}) + \mathrm{P}_u(\text{“calm"}) \tag{4}$$

and

$$\text{arousal}(u) = \mathrm{P}_u(\text{“happy"}) + \mathrm{P}_u(\text{“angry"}) - \mathrm{P}_u(\text{“gloomy"}) - \mathrm{P}_u(\text{“calm"}). \tag{5}$$

The result is a point that lies in the 2×2 square with corners at $(\pm 1, \pm 1)$.

For example, if we use the quote from Maria Sabina, u = "Heal yourself, with beautiful love, and always remember...you are the medicine" the model gives the scores

$$\begin{array}{lll} \bullet\ \mathrm{P}_u(\text{"happy"}) & = & 0.46 \\ \bullet\ \mathrm{P}_u(\text{"angry"}) & = & 0.02 \\ \bullet\ \mathrm{P}_u(\text{"gloomy"}) & = & 0.05 \\ \bullet\ \text{and } \mathrm{P}_u(\text{"calm"}) & = & 0.47 \end{array} \tag{6}$$

The resulting valence and arousal numbers computed from equations (4) and (5) are

$$\begin{array}{lll} \bullet\ \text{valence}(u) & = & 0.87 \\ \bullet\ \text{arousal}(u) & = & -0.04 \end{array} \tag{7}$$

putting the sentiment score in an area that is very positive, and on the calm side of neutral arousal (see Figure 1.4.1).

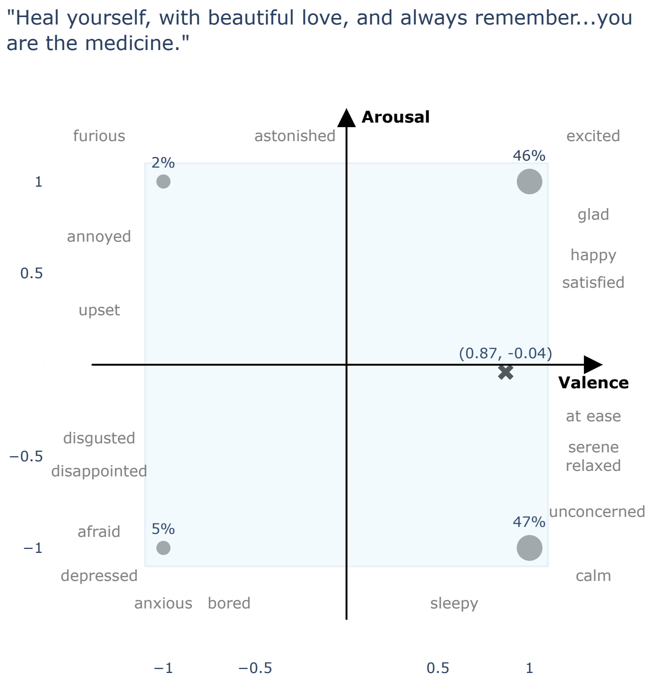

Figure 1: The 2d sentiment plot of a quote by María Sabina. The "X" coordinates are from equation (7), and computed from the corner values in equation (6).

Ultimately, these classes were chosen because the resulting valence and arousal scores showed strong agreement with human scoring when tested on random samples. Other strategies for extracting valence and arousal scores that were less effec-

tive included simply using the classes “positive" (1, 0), “negative" (-1, 0), “aroused" (0, 1), “unaroused" (0, -1), as well as variations on the above.

1.4.2 The Session Sentiment Score

The set of utterances u' of a given speaker (participant, lead therapist, etc.) during a single visit (first preparation, first integration, etc.) was given a probability measure where the measure $p(u)$ of a given utterance u is proportional to the number of words in the utterance $\mathrm{W}(u)$:

$$p(u) = \mathrm{W}(u) \,/\, \sum_{u'} \mathrm{W}(u'). \tag{8}$$

From this, we obtain mean valence and arousal scores in the usual way:

$$\text{valence\_mean} = \mathbf{E}(\text{valence}) = \sum_{u} \text{valence}(u)\, p(u) \tag{9}$$

and

$$\text{arousal\_mean} = \mathbf{E}(\text{arousal}) = \sum_{u} \text{arousal}(u)\, p(u). \tag{10}$$

Figures 1.4.2 and 1.4.2 give a concise visual summary of all the sentiment scores of a speaker during a session, as well as the mean sentiment value itself.

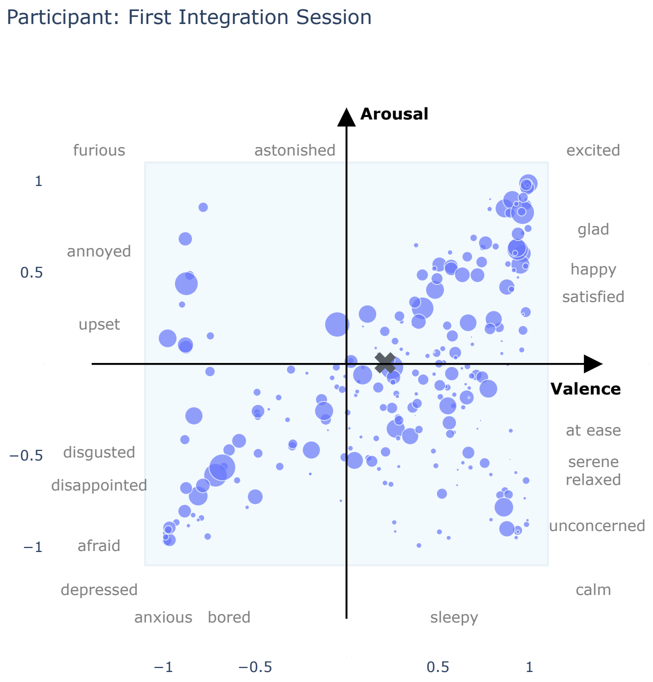

Figure 2: The 2d sentiment plot of a participant's utterances during their first integration session. Each circle represents an utterance, and a circle's size reflects the relative length $p(u)$ of the utterance. The "X" marks the mean sentiment value (valence_mean, arousal_mean) of all the participant's utterances during the session.

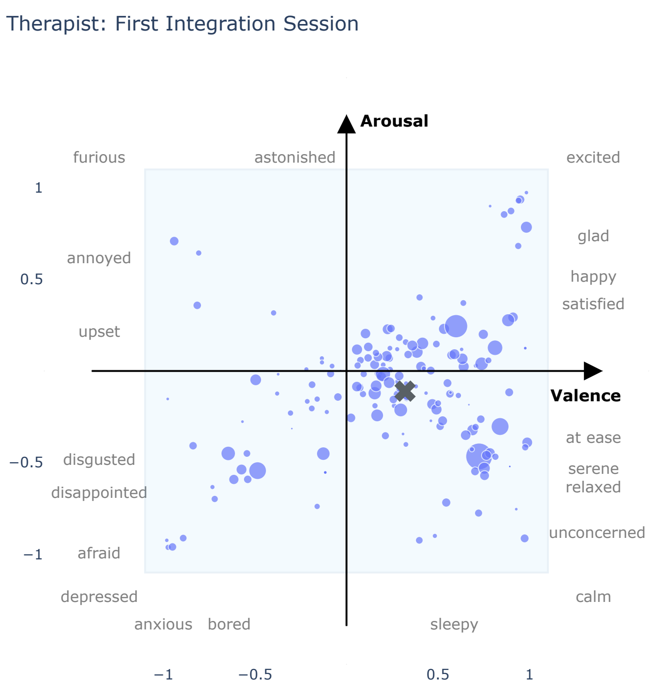

Figure 3: The 2d sentiment plot of a therapist's utterances during the same integration session depicted in figure 1.4.2. Each circle represents an utterance, and a circle's size reflects the relative length $p(u)$ of the utterance. The "X" marks the mean sentiment value (valence_mean, arousal_mean) of all the therapist's utterances during the session.

2 Model Fitting

Two logistic regression models were used to predict whether a participant was a responder or a sustained responder. As noted above, a responder was defined as a participant whose MADRS score 3 weeks post psilocybin administration was reduced by at least 50% relative to their baseline MADRS score, and a sustained responder had at least a 50% reduction in MADRS score at weeks 3, 6, 9, and 12. Both models used the participant's response on the Emotional Breakthrough Inventory (EBI), the treatment dose, and four sentiment metrics from the transcript of the first integration session.

We first fit each of the models to the full data to ensure that the selected models resulted in an adequate fit. We then used leave-one-out (LOO) cross-validation to assess the predictive power of these models. The LOO method fits the model to all participants except one and uses the model to predict the left out participant's responder status. This is repeated for each of the 101 participants and the prediction results are aggregated across all the 101 left-out predictions. This approach simulates the case where a model that was trained on existing data is used to predict the responder status of a new participant.

Treatment was encoded as a categorical variable with values of 0, 1, and 2 for the 1mg, 10mg and 25mg treatment groups, respectively. The four therapy session sentiment scores were normalized to have zero mean and a standard deviation of one. The PCA was fit to the raw EBI scores, as all eight scores have the same scale. The weights from the first principal component were used to transform the raw EBI scores into a single summary score. This summary score as computed is normalized, so required no further treatment.

To ensure no information leaked between the training data and the left-out test participant, all model fitting steps (EBI PCA fit, sentiment score normalization parameter fits, and logistic regression model fit) were performed in the cross-validation loop and thus did not have the advantage of including the left-out participant in any stage of model fitting.

2.1 EBI PCA

The EBI was initially developed as an eight-item questionnaire rated on a 0-100 visual analogue scale (VAS), with two items requiring reverse scoring [6]. This item selection for EBI was derived from interviews of patients after a small trial of psilocybin treatment for TRD [7], followed by expert review. Subsequent PCA-based factor analysis revealed two factors, with the six questions that loaded heavily on the first factor proposed for widespread use [6]. However, given the small sample size in that study, further exploration of the ideal EBI summary score was warranted.

Given our relatively large sample size, we chose to use PCA to summarize the eight EBI items into a single score based on the first principle component, which explained 87% of the variance in the item scores. The weights for this component are shown in Table 1. Our PCA analysis both validates the EBI data in our data set and provides further support to the results of [6] as the positive weights on items $\{1, 2, 3, 5, 6, 8\}$ and negative weights on items {4,7} agree well with the factor scores

that they report.

Note that to avoid information leakage, the EBI PCA was performed within the leave-one-out cross-validation loop, while the illustrative values shown in Table 1 are for a PCA fit to all the data.

Table 1: EBI PCA weights for the first principal component.

Item	Prompt	Weight
EBI05	I had an emotional breakthrough.	0.4313
EBI08	I achieved an emotional release followed by a sense of relief.	0.4227
EBI03	I felt able to explore challenging emotions and memories.	0.4044
EBI06	I was able to get a sense of closure on an emotional problem.	0.3844
EBI02	I experienced a resolution of a personal conflict/trauma.	0.3782
EBI01	I faced up to emotional difficult feelings that I usually push aside.	0.3313
EBI07	I felt emotionally stuck throughout, without a breakthrough.	-0.2619
EBI04	I was resisting and avoiding challenging feelings throughout, without a breakthrough.	-0.0521

2.2 Logistic Regression Model Fit Details

The full logistic regression model fit details are shown in Tables 2 and 3. Consistent with the scatter plots in Figure 3 of the main paper, the model coefficients for the exogenous variables are generally positive, and in the one case of a negative coefficient (participant valence in Table 3), it is not statistically significant. We also note that we did not drop exogenous variables with non-significant coefficients to avoid over-tuning our models and straying from the original hypothesis that integration session data would predict outcome at 3 and 12 weeks.

To further explore the relation between NLP, EBI and treatment, we also fit a model with just treatment that was statistically significant for the 25 mg dose, consistent with results for the full sample of 233 participants [8]. As noted in the conclusion, we propose that the treatment effect on the MADRS outcome is mediated by the positive emotional impact it induces, as measured by the sentiment NLP scores and EBI ratings.

Table 2: Logistic Regression model fit for week 3 responders.

**Dep. Variable:**	responder_wk3	**No. Observations:**	101
**Model:**	Logit	**Df Residuals:**	93
**Method:**	MLE	**Df Model:**	7
**Date:**	Wed, 14 Sep 2022	**Pseudo R-squ.:**	0.5136
**Time:**	14:25:10	**Log-Likelihood:**	-30.679
**converged:**	True	**LL-Null:**	-63.070
**Covariance Type:**	nonrobust	**LLR p-value:**	1.663e-11

	**coef**	**std err**	**z**	**P>|z|**	**[0.025**	**0.975]**
**Intercept**	2.4604	1.744	1.411	0.158	-0.958	5.879
**C(treatment)[T.1]**	-0.3232	0.981	-0.330	0.742	-2.245	1.599
**C(treatment)[T.2]**	1.4213	0.907	1.567	0.117	-0.357	3.199
**pca_ebi0**	0.0123	0.005	2.577	0.010	0.003	0.022
**v4_normalized_arousal_mean_pt**	7.8643	5.696	1.381	0.167	-3.300	19.029
**v4_normalized_valence_mean_pt**	2.6354	2.416	1.091	0.275	-2.101	7.371
**v4_normalized_arousal_mean_th**	14.0501	6.609	2.126	0.034	1.096	27.004
**v4_normalized_valence_mean_th**	4.3393	3.394	1.279	0.201	-2.312	10.991

Table 3: Logistic Regression model fit for sustained responders.

**Dep. Variable:**	responder_sustained	**No. Observations:**	90
**Model:**	Logit	**Df Residuals:**	82
**Method:**	MLE	**Df Model:**	7
**Date:**	Wed, 14 Sep 2022	**Pseudo R-squ.:**	0.4350
**Time:**	14:25:13	**Log-Likelihood:**	-25.445
**converged:**	True	**LL-Null:**	-45.036
**Covariance Type:**	nonrobust	**LLR p-value:**	1.804e-06

	**coef**	**std err**	**z**	**P>|z|**	**[0.025**	**0.975]**
**Intercept**	-0.1261	1.781	-0.071	0.944	-3.617	3.365
**C(treatment)[T.1]**	-0.9520	1.190	-0.800	0.424	-3.284	1.380
**C(treatment)[T.2]**	0.8916	0.965	0.924	0.355	-0.999	2.782
**pca_ebi0**	0.0119	0.006	1.960	0.050	-9.79e-07	0.024
**v4_normalized_arousal_mean_pt**	6.8561	5.654	1.213	0.225	-4.226	17.938
**v4_normalized_valence_mean_pt**	-1.7501	2.747	-0.637	0.524	-7.134	3.634
**v4_normalized_arousal_mean_th**	7.5977	6.163	1.233	0.218	-4.481	19.676
**v4_normalized_valence_mean_th**	4.1433	3.336	1.242	0.214	-2.395	10.682

2.3 Partial Model Predictions

To assess the predictive performance of each of the exogenous variables, we fit various partial models that include subsets of the exogenous variables. The results from leave-one-out cross-validation for these partial models are shown in Tables 4 and 5. Interestingly, EBI and the four NLP measures perform about equally well. While model performance is improved when both are included, it is not substantially improved over either alone, suggesting that these two measures are perhaps driven by the same latent variable. This observation warrants further exploration.

Table 4: Week 3 responder model prediction results.

		Accuracy	MCC	f1	AUC	TN	FP	FN	TP	N
Group	Model									
ALL	EBI	0.772	0.478	0.646	0.831	57	12	11	21	101
	NLP	0.802	0.529	0.667	0.831	61	8	12	20	101
	EBI and NLP	0.802	0.535	0.677	0.877	60	9	11	21	101
	Full	0.851	0.650	0.754	0.877	63	6	9	23	101
1 mg	EBI	0.839	0.392	0.444	0.813	24	1	4	2	31
	NLP	0.774	0.406	0.533	0.793	20	5	2	4	31
	EBI and NLP	0.839	0.451	0.545	0.860	23	2	3	3	31
	Full	0.806	0.380	0.500	0.820	22	3	3	3	31
10 mg	EBI	0.742	0.299	0.429	0.762	20	6	2	3	31
	NLP	0.871	0.523	0.600	0.908	24	2	2	3	31
	EBI and NLP	0.774	0.343	0.462	0.885	21	5	2	3	31
	Full	0.935	0.746	0.750	0.862	26	0	2	3	31
25 mg	EBI	0.744	0.484	0.762	0.833	13	5	5	16	39
	NLP	0.769	0.586	0.743	0.828	17	1	8	13	39
	EBI and NLP	0.795	0.606	0.789	0.886	16	2	6	15	39
	Full	0.821	0.641	0.829	0.886	15	3	4	17	39

Table 5: Sustained responder model prediction results.

Group	Model	Accuracy	MCC	f1	AUC	TN	FP	FN	TP	N
ALL	EBI	0.844	0.475	0.563	0.841	67	5	9	9	90
	NLP	0.800	0.234	0.308	0.817	68	4	14	4	90
	EBI and NLP	0.856	0.522	0.606	0.853	67	5	8	10	90
	Full	0.878	0.610	0.686	0.853	67	5	6	12	90
1 mg	EBI	0.889	0.350	0.400	0.792	23	1	2	1	27
	NLP	0.852	-0.069	0.000	0.694	23	1	3	0	27
	EBI and NLP	0.852	0.250	0.333	0.736	22	2	2	1	27
	Full	0.852	0.250	0.333	0.722	22	2	2	1	27
10 mg	EBI	0.852	-0.080	0.000	0.700	23	2	2	0	27
	NLP	0.889	0.350	0.400	0.880	23	2	1	1	27
	EBI and NLP	0.926	0.460	0.500	0.760	24	1	1	1	27
	Full	0.963	0.693	0.667	0.760	25	0	1	1	27
25 mg	EBI	0.806	0.567	0.696	0.870	21	2	5	8	36
	NLP	0.694	0.286	0.353	0.863	22	1	10	3	36
	EBI and NLP	0.806	0.567	0.696	0.883	21	2	5	8	36
	Full	0.833	0.639	0.769	0.880	20	3	3	10	36

The python code used to compute the sentiment scores and reproduce many of the figures in the paper and associated supplemental materials can be found at `https://github.com/compasspathways/Sentiment2D`.

References

[1] J. Russell, A circumplex model of affect, Journal of personality and social psychology 39 (6) (1980) 1161–1178.

[2] W. Yin, J. Hay, D. Roth, Benchmarking zero-shot text classification: Datasets, evaluation and entailment approach, in: Proceedings of the 2019 Conference on Empirical Methods in Natural Language Processing and the 9th International Joint Conference on Natural Language Processing (EMNLP-IJCNLP), Association for Computational Linguistics, Hong Kong, China, 2019, pp. 3914–3923. doi:10.18653/v1/D19-1404.
URL https://aclanthology.org/D19-1404

[3] M. Lewis, Y. Liu, N. Goyal, M. Ghazvininejad, A. Mohamed, O. Levy, V. Stoyanov, L. Zettlemoyer, BART: Denoising sequence-to-sequence pre-training for natural language generation, translation, and comprehension, in: Proceedings of the 58th Annual Meeting of the Association for Computational Linguistics, Association for Computational Linguistics, Online, 2020, pp. 7871–7880. doi:10.18653/v1/2020.acl-main.703.
URL https://aclanthology.org/2020.acl-main.703

[4] A. Williams, N. Nangia, S. Bowman, A broad-coverage challenge corpus for sentence understanding through inference, in: Proceedings of the 2018 Conference of the North American Chapter of the Association for Computational Linguistics: Human Language Technologies, Volume 1 (Long Papers), Association for Computational Linguistics, 2018, pp. 1112–1122.
URL `http://aclweb.org/anthology/N18-1101`

[5] T. Wolf, L. Debut, V. Sanh, J. Chaumond, C. Delangue, A. Moi, P. Cistac, T. Rault, R. Louf, M. Funtowicz, et al., Huggingface's transformers: State-of-the-art natural language processing (2019).

[6] L. Roseman, E. Haijen, K. Idialu-Ikato, M. Kaelen, R. Watts, R. Carhart-Harris, Emotional breakthrough and psychedelics: Validation of the emotional breakthrough inventory, Journal of Psychopharmacology (2019) 026988111985597–026988111985597`doi:10.1177/0269881119855974`.
URL `http://dx.doi.org/10.1177/0269881119855974`

[7] R. Watts, C. Day, J. Krzanowski, D. Nutt, R. Carhart-Harris, Patients' accounts of increased 'connectedness' and 'acceptance' after psilocybin for treatment-resistant depression, J Humanist Psychol 57 (2017) 520–564. `doi:10.1177/0022167817709585`.
URL `https://www.bmj.com/content/340/bmj.c1468`

[8] G. M. Goodwin, S. T. Aaronson, O. Alvarez, P. C. Arden, A. Baker, J. C. Bennett, C. Bird, R. E. Blom, C. Brennan, D. Brusch, et al., Single-dose psilocybin for a treatment-resistant episode of major depression, New England Journal of Medicine 387 (18) (2022) 1637–1648.
